# Elastic and anelastic relaxation behaviour of perovskite multiferroics II: PbZr_0.53_Ti_0.47_O_3_ (PZT)–PbFe_0.5_Ta_0.5_O_3_ (PFT)

**DOI:** 10.1007/s10853-016-0330-9

**Published:** 2016-09-09

**Authors:** J. A. Schiemer, I. Lascu, R. J. Harrison, A. Kumar, R. S. Katiyar, D. A. Sanchez, N. Ortega, C. Salazar Mejia, W. Schnelle, H. Shinohara, A. J. F. Heap, R. Nagaratnam, S. E. Dutton, J. F. Scott, B. Nair, N. D. Mathur, M. A. Carpenter

**Affiliations:** 1 Department of Earth Sciences, University of Cambridge, Downing Street, Cambridge, CB2 3EQ UK; 2 CSIR-National Physical Laboratory, Dr. K. S. Krishnan Marg, New Delhi, 110012 India; 3 Department of Physics and Institute for Functional Nanomaterials, University of Puerto Rico, PO Box 23334, San Juan, PR 00931-3334 USA; 4 Max Planck Institute for Chemical Physics of Solids, Nöthnitzer Straße 40, 01187 Dresden, Germany; 5 Cavendish Laboratory, University of Cambridge, Madingley Road, Cambridge, CB3 0HE UK; 6 School of Physics and Astronomy, University of St. Andrews, North Haugh, St. Andrews, KY16 9SS UK; 7 Department of Materials Science, University of Cambridge, 27 Charles Babbage Road, Cambridge, CB3 0FS UK

## Abstract

Elastic and anelastic properties of ceramic samples of multiferroic perovskites with nominal compositions across the binary join PbZr_0.53_Ti_0.47_O_3_–PbFe_0.5_Ta_0.5_O_3_ (PZT–PFT) have been assembled to create a binary phase diagram and to address the role of strain relaxation associated with their phase transitions. Structural relationships are similar to those observed previously for PbZr_0.53_Ti_0.47_O_3_–PbFe_0.5_Nb_0.5_O_3_ (PZT–PFN), but the magnitude of the tetragonal shear strain associated with the ferroelectric order parameter appears to be much smaller. This leads to relaxor character for the development of ferroelectric properties in the end member PbFe_0.5_Ta_0.5_O_3_. As for PZT–PFN, there appear to be two discrete instabilities rather than simply a reorientation of the electric dipole in the transition sequence cubic–tetragonal–monoclinic, and the second transition has characteristics typical of an improper ferroelastic. At intermediate compositions, the ferroelastic microstructure has strain heterogeneities on a mesoscopic length scale and, probably, also on a microscopic scale. This results in a wide anelastic freezing interval for strain-related defects rather than the freezing of discrete twin walls that would occur in a conventional ferroelastic material. In PFT, however, the acoustic loss behaviour more nearly resembles that due to freezing of conventional ferroelastic twin walls. Precursor softening of the shear modulus in both PFT and PFN does not fit with a Vogel–Fulcher description, but in PFT there is a temperature interval where the softening conforms to a power law suggestive of the role of fluctuations of the order parameter with dispersion along one branch of the Brillouin zone. Magnetic ordering appears to be coupled only weakly with a volume strain and not with shear strain but, as with multiferroic PZT–PFN perovskites, takes place within crystals which have significant strain heterogeneities on different length scales.

## Introduction

Solid solutions involving the disordered, magnetic III-V perovskites PbFe_0.5_Ta_0.5_O_3_ (PFT), PbFe_0.5_Nb_0.5_O_3_ (PFN) and PbFe_0.5_W_0.5_O_3_ (PFW) with either PbTiO_3_ (PT) or PbZr_1−*x*_Ti_*x*_O_3_ (PZT) have been receiving close attention for their potential as single-phase multiferroic materials [1–13]. Some possess multiferroic properties at room temperature, and magnetic switching of ferroelectric domains has been achieved in PZT–PFT [1, 10]. The wider context relates to opportunities for creating non-volatile memory devices with magnetic read and electrical write operations, among many other possible applications (see for example Refs. [14, 15]). It is likely that the strength of magnetoelectric effects, as well as the dynamics of switching, will be mediated by coupling with strain and hence that ferroelasticity is an important part of the overall multiferroic behaviour. The present work follows from Schiemer et al. [16] on perovskites with compositions in the PZT–PFN solid solution and has, as its primary objective, consideration of the strength and dynamics of elastic strain relaxation mechanisms in the system PZT–PFT.

Given the close similarity in radii of Ta^5+^ and Nb^5+^ (both $0.64 \,\AA$ for octahedral coordination in the tables of Shannon [17]), the initial expectation is that the transformation behaviour and properties of PFT will be similar to those of PFN. With increasing PFT content across the PFN–PFT solid solution, the ferroelectric transition occurs at progressively lower temperatures, from ~390 to ~270 K, but takes on relaxor characteristics [18, 19]. Transformation behaviour at ternary compositions within the system PZ–PT–PFT is similar to that of ternary phases in PZ–PT–PFN, as can be seen by comparing previously published data for the elastic and anelastic properties of [Pb(Zr_0.53_Ti_0.47_)O_3_]_0.6_[PFT]_0.4_ [8] with those for [Pb(Zr_0.53_Ti_0.47_)O_3_]_0.6_[PFN]_0.4_ [16]. The properties of PFT are significantly different from those of PFN, however, and a key factor in the change from ferroelectric to relaxor behaviour appears to be a significant reduction in the strength of coupling with the tetragonal shear strain.

Following an analysis of the spontaneous strain determined from published lattice parameter data for PFT, a binary phase diagram for the binary join Pb(Zr_0.53_Ti_0.47_)O_3_–PFT is proposed here. New elastic and anelastic data obtained by resonant ultrasound spectroscopy from a ceramic sample of PFT are presented, and data from Schiemer et al. [8] for [Pb(Zr_0.53_Ti_0.47_)O_3_]_0.6_[PFT]_0.4_ (PZTFT4) are then reinterpreted in the light of the evidence from PFN [20] and Pb(Zr_0.53_Ti_0.47_)O_3_–PFN [16] that local strain heterogeneity on a mesoscopic length scale is important in these multicomponent, multiferroic perovskites.

## PFT

The structural sequence currently accepted in the literature for PFT is cubic ($Pm{\bar{3}}m$, or $Fm{\bar{3}}m$ if a degree of B-site ordering occurs)–tetragonal (*P*4*mm*)–monoclinic (*Cm*), with reported transition temperatures of ~270 and ~220 K [21–23]. Peak splitting of diffraction maxima in powder diffraction patterns from the tetragonal phase has not yet been observed, however, indicating that any distortion from cubic lattice geometry is at or below the limit of resolution for conventional powder X-ray diffraction. A more sensitive indicator of strain is birefringence. Single crystals show very low birefringence in the stability field of the cubic structure (~10^−4^; [23–25]), which Brixel et al. [24] attributed to the influence of defects and which Geddo Lehmann and Sciau [23] discussed in terms of precursor effects ahead of the transition to the tetragonal structure. A small and continuous change in birefringence occurs in the vicinity of 270 K [23] or ~248 K [24] but even then only reaches ~10^−3^ on cooling to 215 K [24]. In neither study was any evidence for the development of ferroelastic twins observed. By way of contrast, the birefringence increases steeply to 0.03–0.06 below a weakly first-order transition at 210 K (heating) or 205 K (cooling), and this is accompanied by the development of ferroelastic twinning on an optical scale [24].

**Figure 1:**
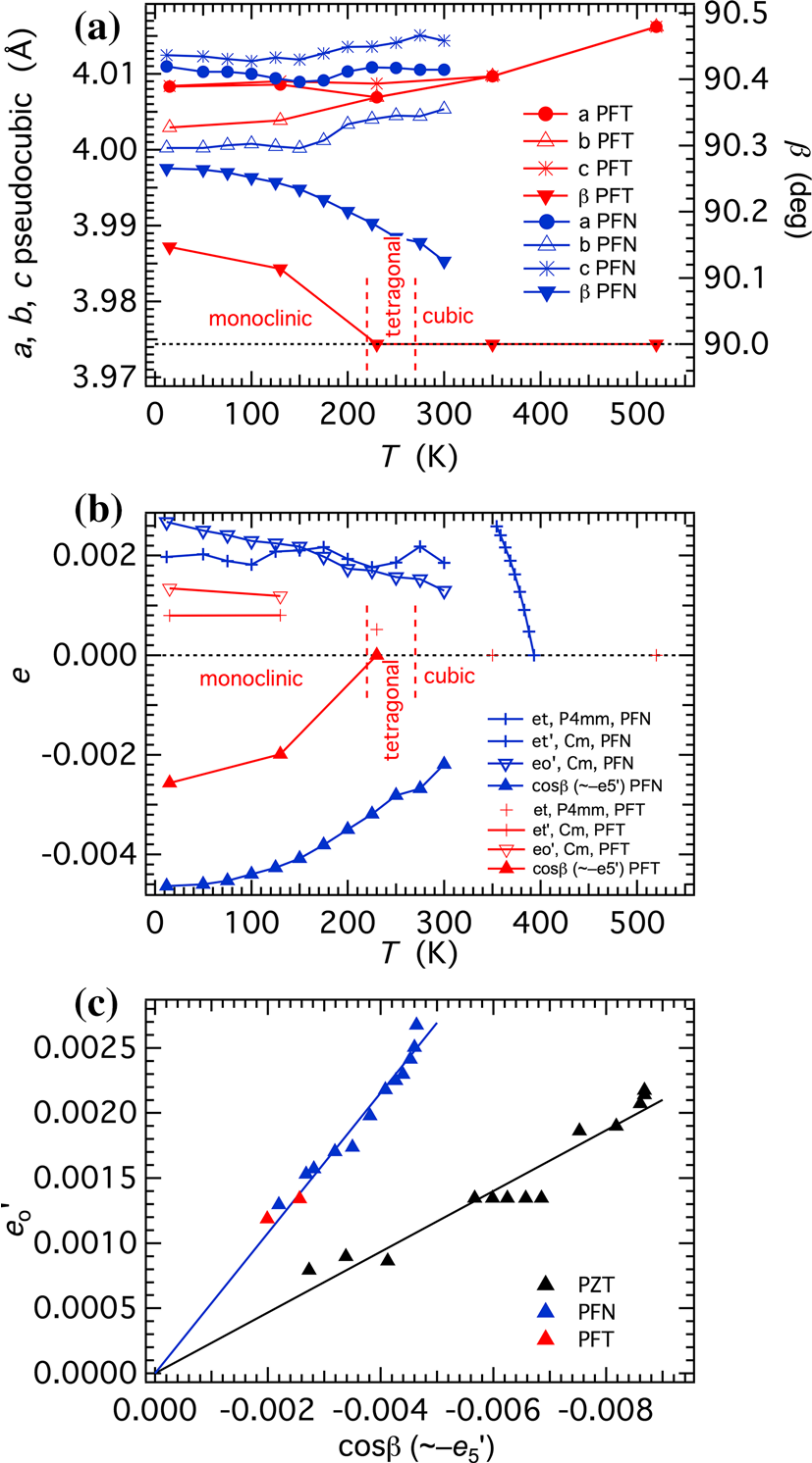
Strain analysis for PFT, based on lattice parameter data from Lampis et al. [21]. **a** Lattice parameters, with the setting of *a* and *c* chosen so as to give *β* > 90°. Quoted experimental uncertainties are smaller than the size of the symbols. Also shown are lattice parameters for the monoclinic structure of PFN from Singh et al. [29]. **b** Symmetry-adapted shear strains calculated from the lattice parameter data in (**a**). **c** Shear strains of PFT are smaller than those of PFN but display the same linear interdependence, which is different from that shown by Pb(Zr_0.52_Ti_0.48_)O_3_, labelled PZT (following [16])

Data from the literature for lattice parameter variations allow comparison of the spontaneous strains accompanying the transitions in PFT with those of PFN. They show that the pattern of variations is comparable, but that deviations from cubic geometry are smaller (Fig. 1). The primary data from Lampis et al. [21] are given in Fig. 1a and values of the symmetry-adapted shear strains, $e_{\rm{t}}^{'}$, $e_{\rm{o}}^{'}$ and $e_{5}^{'}$, calculated from them using the expressions given in Carpenter et al. [20] and Schiemer et al. [16], are given in Fig. 1b. Values of the reference parameter, *a*_o_, were calculated as *a*_o_ = (*a*_pc_ *b*_pc_ *c*_pc_)^1/3^, where the subscript pc indicates the pseudocubic parameters of the tetragonal and monoclinic structures. The maximum shear strains are less than 0.003 (Fig. 1b). Values smaller than 0.001 are probably below the limit of realistic experimental uncertainties.

The monoclinic structure has three order parameter components, *q*_1_ = *q*_2_ ≠ *q*_3_, which are expected to scale with the three shear strains according to $e_{\rm{t}}^{'} \; \propto q_{3}^{2} - q_{1}^{2}$, $e_{\rm{o}}^{'} \; \propto \;q_{1}^{2}$, $e_{5}^{'} \; \propto \;q_{1} q_{2}$. In combination, $e_{0}^{'}$ and $e_{5}^{'}$ fall on the same trend as for PFN (Fig. 1c) and the structural evolution of the two phases must therefore be closely similar. As with PFN, however, it is possible also to fit the diffraction patterns for the low-temperature structure using rhombohedral symmetry. Lampis et al. [21], Nomura et al. [26] and Ivanov et al. [27] noted that the lattice geometry of their monoclinic structure is pseudo-rhombohedral. The *R*3*m* structure has *q*_1_ = *q*_2_ = *q*_3_ and the shear strain e_4_ (≈cos *α*) would be expected to scale with $q_{1}^{2}$ Rhombohedral lattice angles, *α* from Nomura et al. [26] and Ivanov et al. [27] reach 89.89° and 89.96°, respectively, giving maximum values of |cos *α*| as ~0.002 and ~0.001. Shear strains calculated from the lattice parameter data for tetragonal and monoclinic structures given by Raevski et al. [22, 28] are all smaller than 0.001.

The ferroelectric transition in both PFN and PZT is accompanied by a small, positive volume strain [16, 20], as is relaxor ferroelectric ordering in Pb(Mg_1/3_Nb_2/3_)O_3_ [30]. Consistent with this, the unit cell volume data for PFT in Raevski et al. [22] and the interplanar spacing *d*_400_ of PFT [23] also increase with falling temperature below ~270 K. Although splitting of the *d*_400_ spacing below ~200 K shown by Geddo Lehmann and Sciau [23] appears to be continuous, these authors reported that the tetragonal and monoclinic phases coexist between 200 and 220 K.

Relaxor character for PFT is revealed by a broad maximum in the dielectric constant at a temperature, *T*_m_, in the vicinity of 250 K which varies with frequency in both pure and Li-doped samples [18, 19, 28, 31–42]. This dispersion is described using the Vogel–Fulcher representation of freezing behaviour

1 $$ \nu = \nu_{\rm{o}} \exp \left( {\frac{{ - E_{\text{a}} }}{{{\text{k}}_{\text{B}} (T_{\text{m}} - T_{\text{VF}} )}}} \right), $$

where *ν* is the measurement frequency for a given value of *T*_m_, *ν*_o_ is an attempt frequency, *E*_a_ is an activation energy, k_B_ is the Boltzmann constant and *T*_VF_ is the zero frequency freezing temperature. As shown in Table 1, values for the fit parameters from the literature are scattered. Gusev et al. [42] also reported that one of their samples had a difference of not more than 0.5 K for *T*_m_ measured in the range 10^3^–10^6^ Hz, which is very close to being independent of frequency. The absence of a unique Vogel–Fulcher parameterisation is presumed to reflect differences between samples arising from different methods of synthesis, such as grain size and B-site cation order. In particular, increasing B-site order correlates with an increase in *T*_m_ values and a reduction in their frequency dependence [34]. The data of Gusev et al. [42] also show a correlation between increasing *T*_m_ and decreasing width of peaks in the dielectric permittivity. Presumably, in the limit of complete order, PFT crystals would have a more typically classical ferroelectric transition at ~270 K. Dielectric loss (tan *δ*) measurements show scatter between samples, but a typical form has increases in loss coinciding with the onset of freezing, frequency-dependent maxima and a tail of decreasing loss extending to lower temperatures (e.g. [18, 31, 33, 41, 43]). An additional loss mechanism identified on the basis of peaks in tan *δ* at temperatures within the stability field of the cubic phase can be described using an Arrhenius relationship with an activation energy of 0.9 ± 0.05 eV, and is consistent with conductivity [33].

**Table 1:** Fit parameters from the literature for Vogel–Fulcher freezing (Eq. 1) for the temperature and frequency dependences of the maximum of the real part of the dielectric response of PFT to an AC electric field

Reference	*T*_m_ (K) at ~100 kHz	*E*_a_ (eV)	*ν*_0_ (Hz)	*T*_VF_ (K)
Raevskii et al. [33]	~290	0.12	0.5–1 × 10^11^	170
Bharti et al. [38]	~265	0.46	8.5 × 10^12^	256
Bharti et al. [37]	~265	0.075	2.3 × 10^12^	254
Raevski et al. [28] and Raevskaya et al. [43] (Li-doped sample)	~240	0.018	5 × 10^11^	217
This study	~235	~0.01	~10^5^–10^9^	223

Among all the published dielectric data for PFT, there is no overt evidence for an anomaly which might be associated with the first-order transition near 210 K. Heating in zero field after cooling in an electric field of 5 kV cm^−1^ reveals a frequency-independent shoulder in the dielectric permittivity and tan *δ* at 200 K, which may be this transition, but the development of local ferroelectric order seems to be determined predominantly through the Vogel–Fulcher freezing interval. There is a broad excess in the heat capacity between ~230 and ~330 K [42, 44, 45] that, by analogy with PMN [46], is expected for diffuse, relaxor behaviour. There is a small excess heat capacity associated with the transition at ~205 K, but it appears that this is not driven by a change in ferroelectric polarisation.

The magnetic behaviour of PFT is closely similar to that of PFN. Reported values of the Néel temperature, *T*_N_, are generally between ~120 and 180 K [28, 31, 34, 35, 40, 47–49]. Variations between 160 and 230 K can be induced by varying the annealing temperature used during synthesis [42]. Differences in the degree of B-site order may again be a contributory factor in this context, since increasing order correlates with lowering of *T*_N_ [28, 31, 34]. The low-temperature magnetic structure was originally characterised as ferrimagnetic [47], with small opening of hysteresis loops [22, 40]. Modelling also favours a ferrimagnetic ground state [50].

There seems to be acceptance that additional magnetic anomalies below ~ 10–15 K are due to spin glass behaviour [28, 35, 48, 49, 51–53]. A difference from PFN is that, consistent with the calculation of a possible second Néel point at 48 K by Lampis et al. [52], Martinez et al. [40] proposed another magnetic transition at ~55 K in PFT to account for an anomaly in the temperature dependence of the magnetisation observed in both field-cooled and zero field-cooled measurements. Chillal et al. [49] suggested that some canting of Fe moments occurs below ~50 K. The sample described by Martinez et al. [36, 40] not only displayed a well-defined magnetic hysteresis loop at room temperature but also contained pyrochlore as an impurity phase.

In summary, the strain evolution of PFT can be described as the development of a small positive volume strain continuously with falling temperature below ~270 K, followed by a small rhombohedral shear strain accompanying a weakly first-order transition at ~210 K. Antiferromagnetic ordering in PFN couples with a small (<0.001) negative volume strain (data of Singh et al. [54] in Carpenter et al. [20]) and the same must be expected for PFT. If there is a shear strain coupled with the antiferromagnetic ordering, it amounts to less than ~0.0005 if the changes in the monoclinic *β* angle below 180 K reported by [28] and [22] are taken at face value. Changes in elastic properties are expected to be correspondingly small but the only data in the literature for PFT appear to be measurements of longitudinal acoustic velocity through pure and Li-doped ceramic samples [41, 55]. These show softening by ~50 % with falling temperature from ~425 K to a rounded minimum at ~190 K, followed by a smooth stiffening trend between a trend of softening and then stiffening with falling temperature. The changes in velocity are accompanied by more complex variations in attenuation which have a rounded minimum at ~270 K, a broad peak extending from ~270 to ~50 K with its maximum at ~140 K, followed by a small peak at ~35 K [55, 56].

## PZ–PT–PFT

There are only limited published data available for establishing phase relationships in the ternary system PbZrO_3_–PbTiO_3_–PbFe_0.5_Ta_0.5_O_3_ (PZ–PT–PFT), but the topology is not expected to be significantly different from that of PZ–PT–PFN. Curie temperatures estimated from the temperatures at which the dielectric constant has its maximum value reduce smoothly between PT and PFT [2, 7, 9, 28, 48, 57], and powder X-ray diffraction patterns confirm that the transition from cubic to tetragonal structures at room temperature occurs between 90 and 80 % PFT [2, 28]. Curie temperatures reported for the PZ–PFT join show an irregular variation at PZ-rich compositions, which is presumably due to a limited stability field for the *Pbam* structure, but are otherwise similar [57]. The rhombohedral–rhombohedral transition of PZT extends into the ternary system at PZ-rich compositions [58]. The overall topology for the ternary diagram at room temperature is expected to be closely similar to that shown in Schiemer et al. [16] for PZ–PT–PFN except for a small stability field for the cubic structure at PFT contents greater than ~85 %.

**Figure 2:**
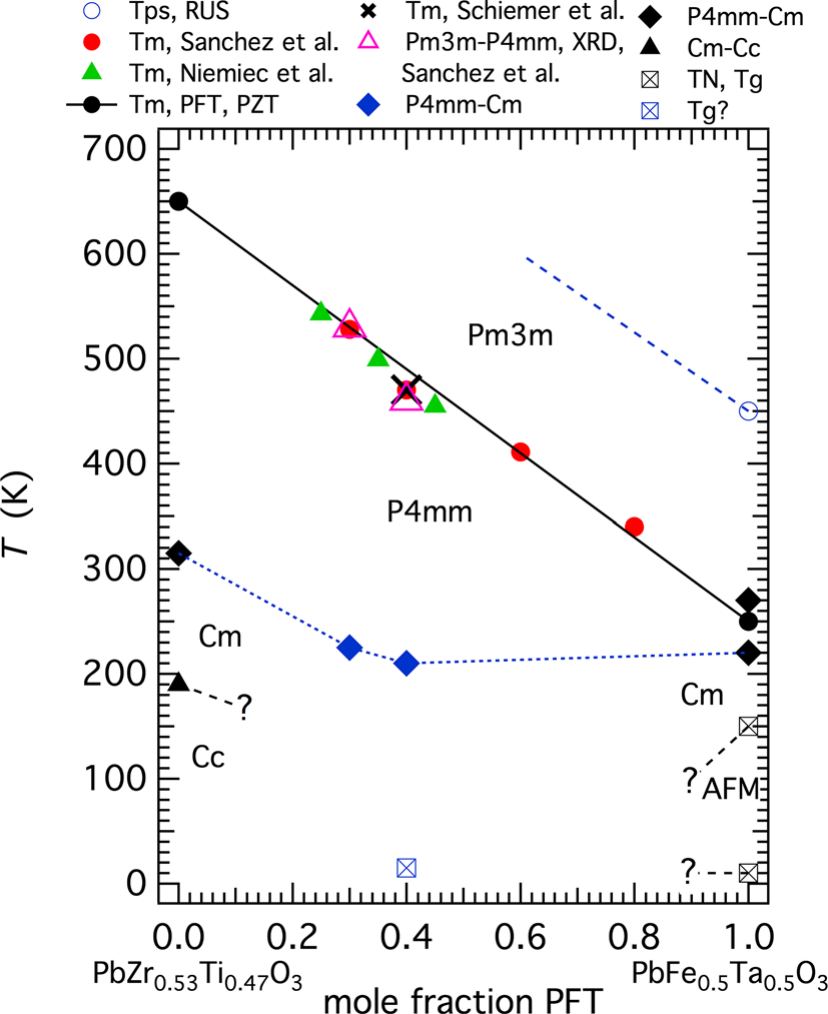
Phase diagram for samples close to the binary join Pb(Zr_0.53_Ti_0.47_)O_3_–PFT. Location of the ferroelectric transition is largely based on determinations of *T*_m_ for ceramic samples [5, 8, 61], together with some X-ray diffraction (XRD) data [12]. The* straight line* shown is a guide to the eye for a linear trend between 650 K (PZT) and 250 K (PFT). *T*_ps_ is the approximate temperature for the onset of precursor softening from RUS measurements. *Dashed lines* are speculative trends for the effects of composition

A cross section for the binary join Pb(Zr_0.53_Ti_0.47_)O_3_–PFT is given in Fig. 2. This shows a linear composition dependence for values of *T*_m_ between ~650 K for PZT and ~250 K for PFT. The expected sequence of transitions in the PZT end member is $Pm{\bar{3}}m$–*P*4*mm*–*Cm*–*Cc*, with transition temperatures ~650, ~315 and ~190 K [59, 60]. The transition temperatures shown for $Pm{\bar{3}}m$–*P*4*mm*–*Cm* in PFT are the values reported from diffraction experiments, 270 and 220 K [21, 23]. Sanchez et al. [12] identified the structural sequence at intermediate compositions as being tetragonal–orthorhombic instead of cubic–tetragonal, but this is most likely due to incorrect indexing of powder diffraction patterns. They placed the first transition at 460 K for a sample with 40 % PFT, which is within experimental uncertainty of *T*_m_ from dielectric measurements. They also stated that there is a further transition to a rhombohedral or monoclinic structure below ~250 K, though without showing the dielectric data on which this conclusion was based. They placed the first transition at ~530 K in a sample with 30 % PFT, based on Raman spectroscopic observations, which is also within experimental uncertainty of *T*_m_ for that composition.

**Figure 3:**
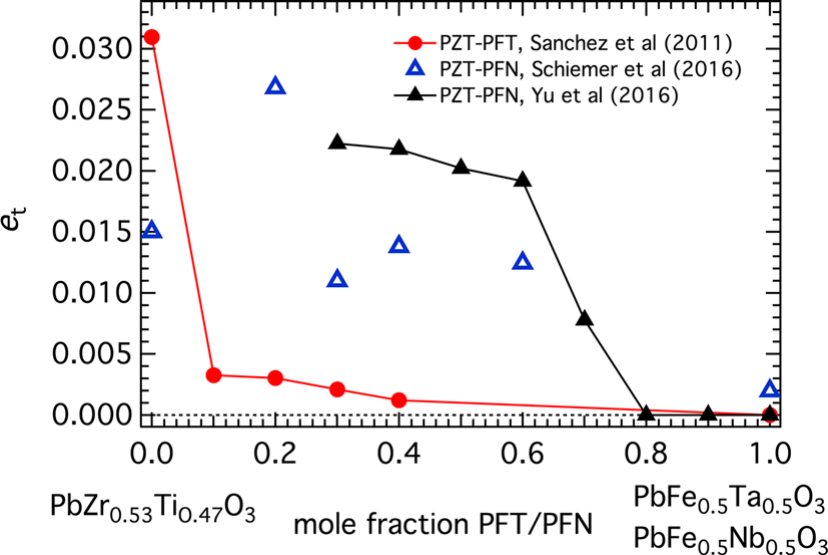
Variation of the tetragonal shear strain, *e*_t_, at room temperature calculated from the lattice parameters given in Table 1 of Ref. [62] for PZT-PFT and from Ref. [16] or Ref. [63] for PZT-PFN

As already indicated, lattice parameter data in Sanchez et al. [12] appear to be for incorrectly assigned structures and there are as yet, no other published data for variations with temperature at intermediate compositions across the Pb(Zr_0.53_Ti_0.47_)O_3_–PFT binary join. However, the magnitudes of the tetragonal shear strain, *e*_t_, can be obtained as a function of composition at room temperature from lattice parameters obtained assuming tetragonal geometry [62]. These are given in Fig. 3 and were calculated as above for the reference parameter, *a*_o_, and according to the expressions in Carpenter et al. [20] for *e*_t_. The remarkable feature is an order of magnitude drop for *e*_t_, in comparison with Pb(Zr_0.53_Ti_0.47_)O_3_, at all intermediate members of the solid solution, which contrasts sharply with the smooth variation of the cubic–tetragonal transition temperature.

Intermediate members of the binary solid solution prepared for the studies of Sanchez and co-workers [8, 61, 62] all showed a small opening in magnetic hysteresis loops, signifying some component of ferromagnetism at room temperature, but samples prepared by Skulski et al. [64] do not. It is notoriously difficult to synthesise pure, homogeneous ceramic samples of ternary PZ–PT–PFN and PZ–PT–PFT perovskites and, even when the diffraction patterns appear to indicate the absence of secondary phases, a very minor magnetic impurity could still contribute to the observed magnetic signals. Anomalies in magnetic susceptibility below ~15 K, observed in both field-cooled and zero field-cooled conditions from a sample of PZTFT4 suggest, at least, that the spin glass-like behaviour of PFT extends into the solid solution, but this has not been fully characterised.

## Sample preparation and characterisation

A ceramic sample of PbFe_0.5_Ta_0.5_O_3_ was prepared in the manner described already by Martinez et al. [36, 40], except that the final sintering at 1050 C was for 10 h instead of 15. Synthesis of the sample of PZTFT4 has been described by Schiemer et al. [8], and the same methodology was used for PZTFT3.

The average composition of the PFT sample, based on 11-point analyses obtained for the final ceramic sample using a Cameca SX-100 electron microprobe, was Pb_0.962±0.004_Zr_0.482±0.007_Ta_0.487±0.004_W_0.037±0.001_O_3_. No impurity phases were detected in backscattered electron images, but some local compositional heterogeneity was seen in elemental maps using a Zeiss UltraPlus FEGSEM. The trace amount of W may have come from the WC ball mill. Schiemer et al. [8] have previously given the composition of the PZTFT4 sample as PbZr_0.30_Ti_0.17_Fe_0.17_Ta_0.27_O_3_.

The X-ray powder diffraction pattern reported for PZTFT4 by Schiemer et al. [8] did not contain evidence of secondary phases. Lattice parameters from tetragonal indexing of diffraction patterns from both PZTFT3 and PZTFT4 are given in Ref. [62]. An X-ray powder diffraction pattern obtained with a Bruker D8 Advance Bragg–Brentano diffractometer from the PFT sample contained weak additional peaks showing the presence of minor impurity phases. The refined lattice parameter, fit with DIFFRACplus TOPAS™ software supplied by Bruker, was *a* = $4.0054(4) \,\AA$.

## Experimental methods

RUS measurements were made on half of a disc (radius ~7 mm, thickness 0.85 mm) of polycrystalline PFT with mass 0.1082 g, using low- and high-temperature equipment that has been described elsewhere [30, 65–67]. Datasets were collected in both cooling and heating sequences with individual spectra containing 65,000 data points in the frequency range 100–1200 kHz for low temperatures (5 K steps, 5–300 K) and 50–1200 kHz for high temperatures (10 K steps, 294–705 K). A 20-min settle time was allowed for thermal equilibration at each set point. Details of the data collection for PZTFT4 were given in Schiemer et al. [8]. Primary spectra were analysed offline using the software package IGOR (Wavemetrics). Selected peaks were fit with an asymmetric Lorentzian function to give their frequency, *f*, and width at half maximum height, Δ*f*. The inverse mechanical quality factor, *Q* ^−1^, is a measure of acoustic loss and is given by Δ*f/f*. Individual resonance modes are dominated by shearing motions so that *f* ^2^ scales closely with the shear modulus, *G*, and has only a small dependence on the bulk modulus, *K*, in most cases.

Magnetic properties were measured on the RUS sample using a Quantum Design MPMS XL SQUID magnetometer in the Department of Engineering of the University of Cambridge (SQUID 1). Our previous investigation had shown that if the ferromagnetism is to be properly understood, high-temperature magnetic measurements are also required. The measurement protocol for susceptibility was unconventional and designed to measure possible coercivity effects, using the residual field of the magnet: ZFC, 300–5 K in the residual field of the magnet followed by collection from 5 to 300 K in the residual field of the magnet; FC, 300 to 5 K in 20 kOe field followed by collection from 5 to 300 K in the residual field of the magnet. While this allows low coercivity components to be examined, the residual field’s magnitude and direction are not known and may be opposite to that applied during field cooling, leading to possible jumps in the measured moment when the coercive field of any remnant ferromagnetism drops below the applied field. Hysteresis loops were measured at fixed temperatures of 5, 30, 130, 160 and 300 K between −50 and 50 kOe on the same magnetometer.

A second Quantum Design magnetometer, in Dresden (SQUID 2), was used to measure magnetisation from 5 up to 700 K using a more conventional zero field-cooled–field-cooled sequence, with temperatures up to 300 K measured in a cryostat and above 300 K measured in a furnace. The sequence of measurements on two small pieces of the RUS sample, weighing 0.0186 g in total, comprised cooling from 700 K in the residual field of the magnet, followed by measurement of the moment in 0.05 kOe applied field during heating (the ZFC measurement), followed again by measurement in 0.05 kOe during cooling (the FC measurement).

A first-order reversal curve (FORC) diagram was obtained at room temperature from a piece of the RUS sample with mass 0.0391 g using a Princeton Measurements Company's alternating gradient magnetometer manufactured by Lake Shore Cryotronics, as described by Schiemer et al. [16]. FORC diagrams are obtained by plotting the FORC distribution, which is the mixed second derivative of the magnetisation with respect to the reversal and measurement fields [68, 69].

Low-temperature impedance measurements were made on a wedge-shaped piece of the RUS sample with an Agilent 4294A Impedance Analyzer. The fragment was mounted in a bespoke cryogenic probe lowered into liquid nitrogen to achieve a measurement temperature range of 100–420 K. Frequency sweeps involved 134 points in the range from 1 to 100 kHz and a sweep was completed every ~0.1 K both during heating and during cooling.

High-temperature dielectric measurements were made on the same sample as had been used for low-temperature dielectric measurement in the same horizontal Netzsch furnace as used for the high-temperature RUS measurements. Electrodes were created on opposite sides using high-temperature silver paste and these were connected by platinum wires to coaxial cables outside the furnace. Data were collected in both heating and cooling cycles, with a temperature ramp of 0.2 K min^−1^ and each measurement taking approximately 10 s. The capacitance is not calibrated as there is a parasitic component expected due to some unshielded length of wire.

## Results

### PFT

**Figure 4:**
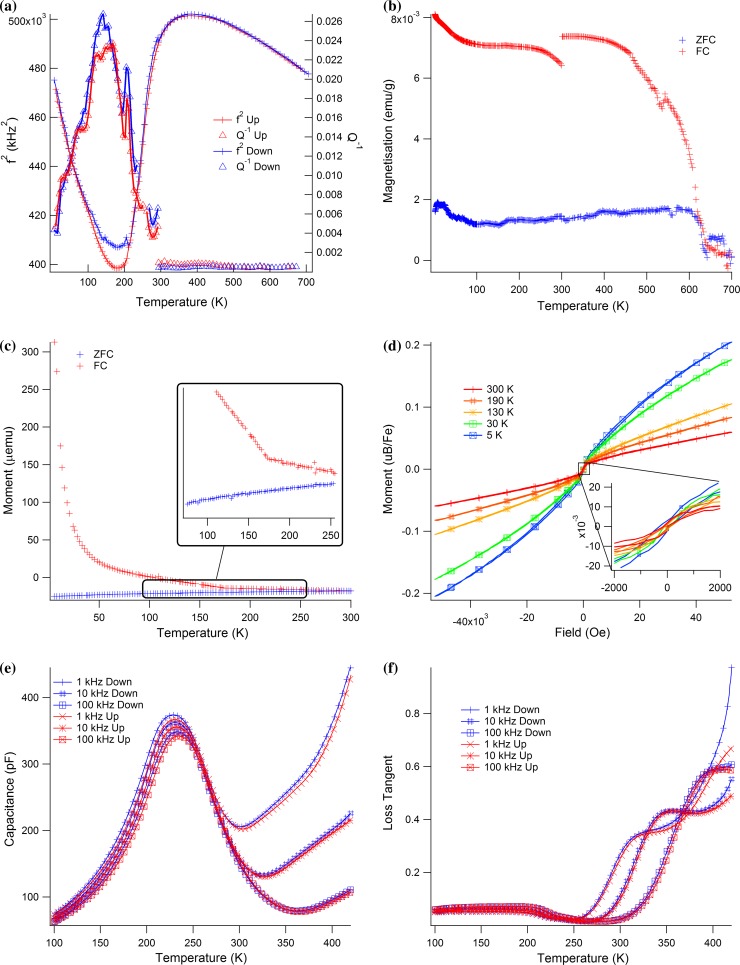

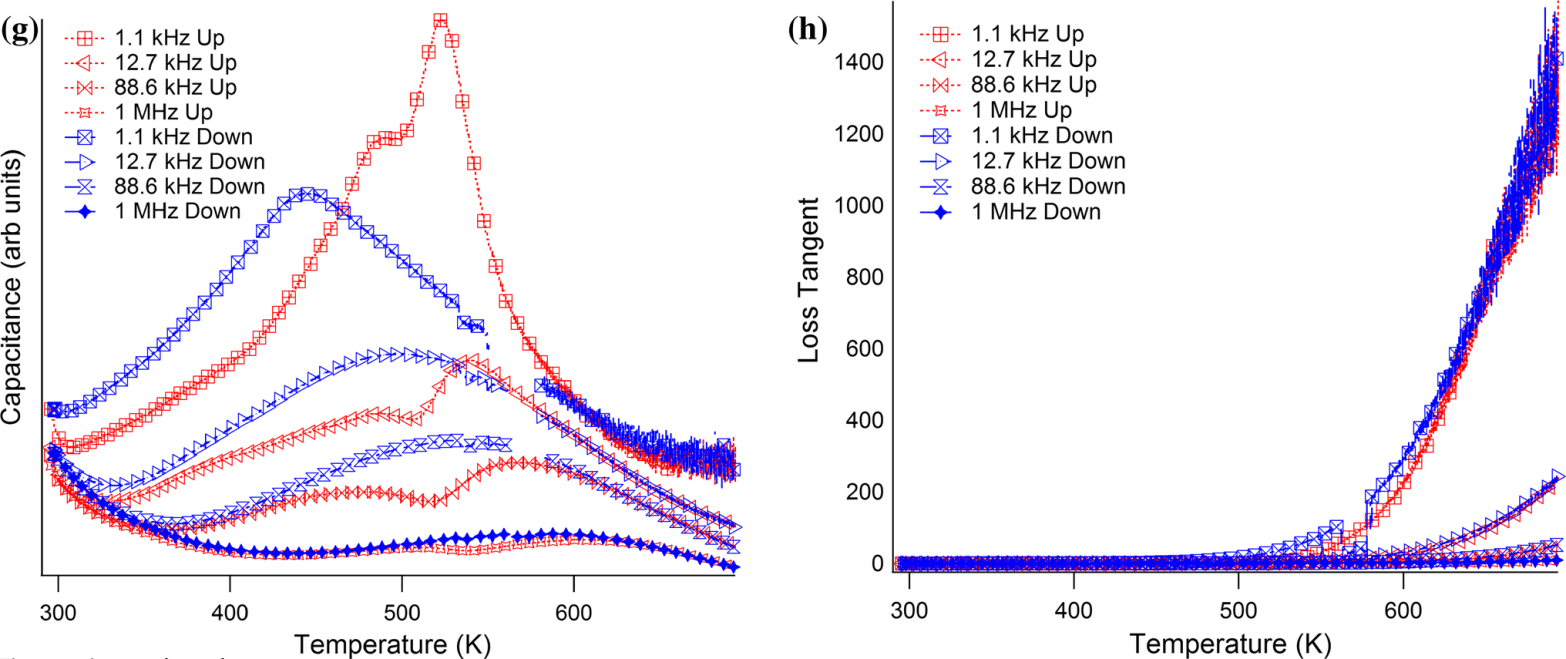
RUS, magnetic moment and dielectric data for PFT. **a** RUS. Results from fitting a low-temperature resonance peak with frequency 170 kHz at room temperature and a high-temperature peak frequency 700 kHz at room temperature. *f* ^2^ data are scaled to match at room temperature, while raw data are given for *Q* ^−1^. The break in values of *Q* ^−1^ at room temperature arises because the sample sits directly between the piezoelectric transducers in the low-temperature instrument but the transducers are outside the furnace in the high-temperature instrument and are separated from the sample by alumina buffer rods. **b** Temperature dependence of magnetic moment as measured in a field of 0.05 kOe (SQUID 2). *Red crosses* are for field-cooled data,* blue* for zero field-cooled (cooling in residual field of instrument, followed by measurement in heating sequence, then cooling in 0.05 kOe field and repeat measurements during heating). The anomaly at 300 K is an artefact and the data appear noisy because the signal was very weak. **c** Magnetic moment data (SQUID 1) showing some divergence between FC and ZFC conditions at all temperatures, but increasing in particular below ~170 K where there is a break in slope of the FC data. **d** Hysteresis loops (SQUID 1): these all have small opening, signifying the presence of some ferromagnetic component at all temperatures from 5 to 300 K (one marker per 10 data points). **e** The low-temperature capacitance data have a broad maximum near 230 K which varies in position and amplitude slightly with changing frequency. Steeply increasing values occur at higher temperatures, with a much stronger frequency dependence. **f** Dielectric loss (tan *δ*) corresponding to the capacitance measurements in **d**: two sets of peaks are seen with different dependence on frequency, weak dependence just above 200 K and much stronger dependence at higher temperatures (one marker per 50 data points). **g** The high-temperature capacitance has two broad frequency-dependent maxima on heating and one on cooling. **h** Dielectric loss (tan *δ*) corresponding to the capacitance measurements in **g** the data are dominated by high losses associated with conductivity at high temperature (one marker per 300 data points)

The RUS data for *f* ^2^ (Fig. 4a) initially show the normal pattern of elastic stiffening (increasing shear modulus) with falling temperature from ~700 K. The trend to softening with further lowering of temperature starts at about 450 K and this becomes much steeper below ~300 K. There is a rounded minimum at ~180 K, with some hysteresis between heating and cooling of the absolute values of *f* ^2^ in the interval ~215–100 K. This is followed by a steep recovery of *f* ^2^, with no sign of levelling off in the trend as *T* → 0 K. The maximum observed softening reaches ~20–25 %. *Q* ^−1^ values remain low at high temperatures but then start to increase below ~280 K. There is a sharp maximum at ~215 K and then a broad peak centred on ~145 K, followed by a continuous decline to the lowest temperatures.

From the evidence of magnetisation data from SQUID 2 (Fig. 4b), weak ferromagnetism appears to extend up to a Curie temperature of ~630 K. It is not yet possible to confirm whether this is intrinsic to the PFT, associated with grain boundaries and other defects, or due to some magnetic impurity. The main features of the magnetic susceptibility (Fig. 4c) are a divergence between the re-scaled FC and ZFC data at all temperatures, a paramagnetic-like trend at low temperatures, although non-linear in 1/*χ* with a non-zero intercept, and a clear break in slope in the FC data (but not in the ZFC data) at around 170 K. The break in slope of the FC data is near to the previously reported Néel temperature of PFT (180 ± 5 K, [40]). A very small hysteresis and saturation with a low coercive field are shown in Fig. 4d, with a noticeable curving of higher field data towards saturation at 5 and 30 K.

**Figure 5:**
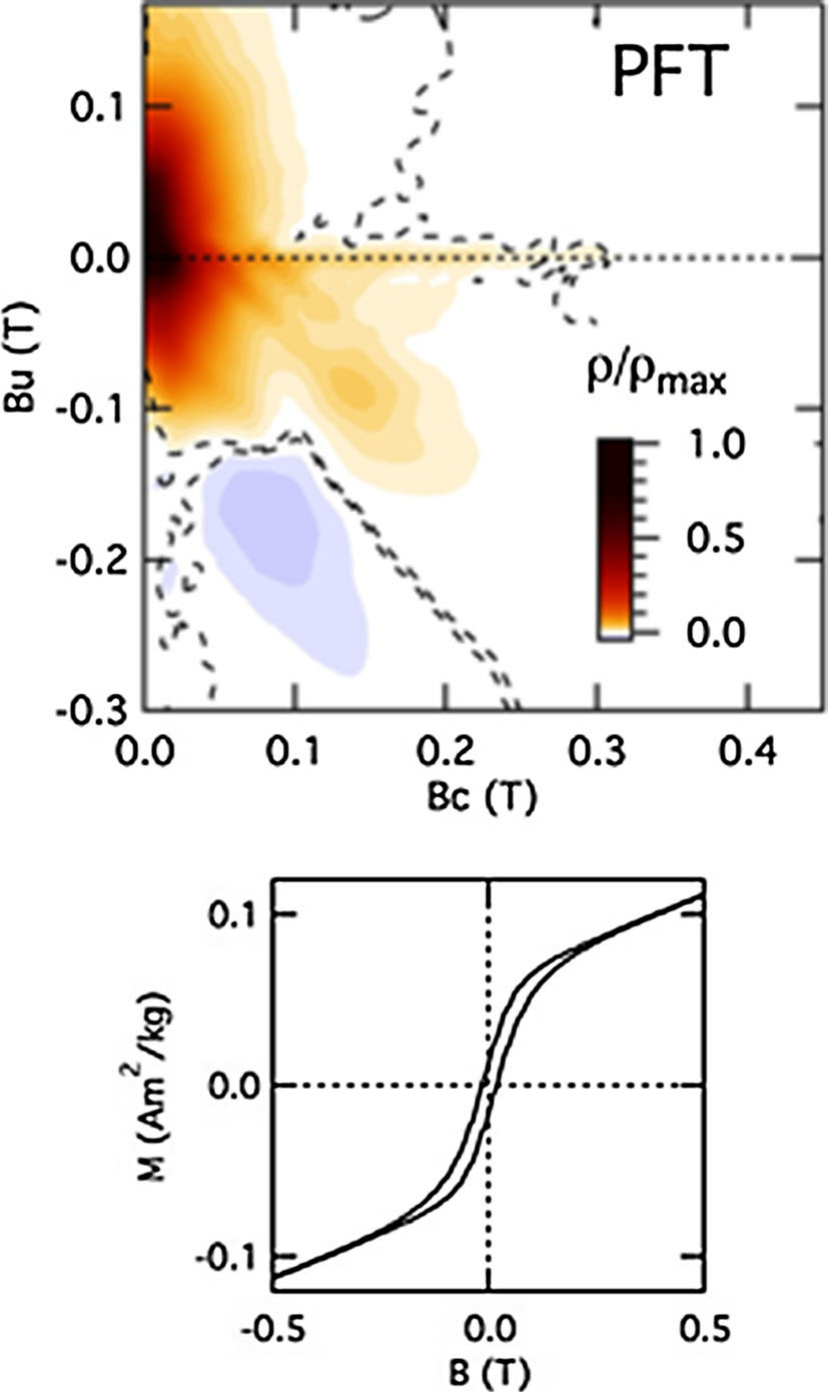
Princeton magnetometer data collected at room temperature. The FORC diagram for PFT exhibits a complex peak structure suggestive of strong magnetostatic interactions between cuboidal ferromagnetic grains that create a net positive mean interaction field. The *dashed line* delimits regions of the FORC distribution (*ρ*) significant at the 0.05 level. The* hysteresis loop* shows narrow opening

The small openings of the hysteresis loops (Figs. 4d, 5) and the separation of FC/ZFC curves are consistent with the view that there is some weakly ferromagnetic component in the sample up to room temperature. This is much weaker than in the previously studied PZT–PFN samples [16, 20], however, and it gives rise to a different pattern of intensity in the FORC diagram, with a positive feature with higher intensity at *B*_c_ values <0.1 T, which spreads vertically along the *B*_u_ axis (Fig. 5). A second positive feature extends along *B*_c_ as a narrow ridge up to coercivities of 0.3 T. A third feature is the pairing of the stronger positive and weaker negative areas along the second quadrant diagonal, at *B*_c_ values >0.05 T and *B*_u_ values < −0.05 T. This complex FORC landscape is akin to that of strongly interacting cuboidal magnetic particles that produce a net positive mean interaction field [70].

**Figure 6:**
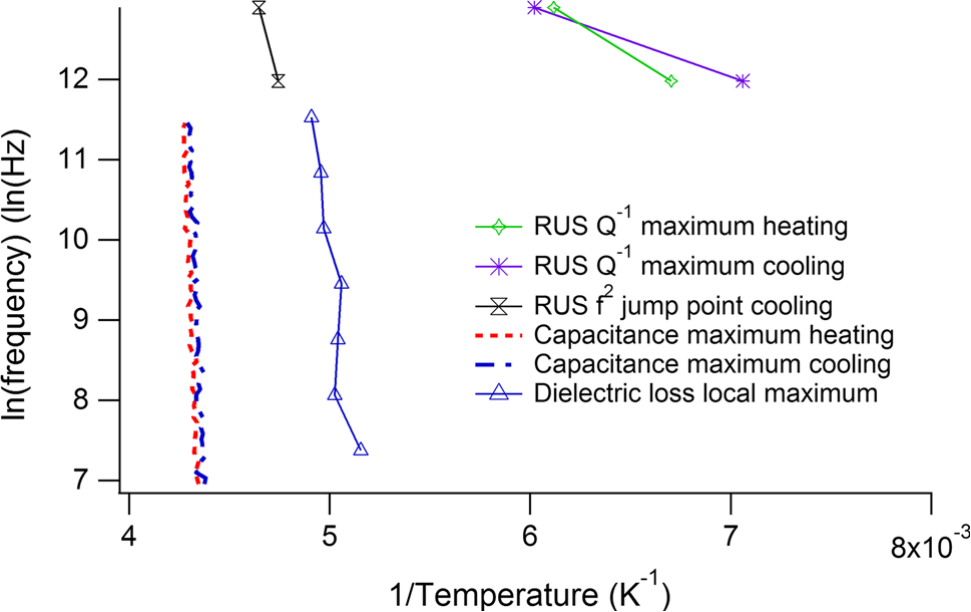
Arrhenius plot for the temperatures at which maxima in capacitance and tan *δ* occur, together with selected anomalies from the RUS data. The dielectric data have only weak dependence on frequency. The temperature at which a small jump in *f* ^2^ occurs is close to temperatures of the dielectric anomaly and is clearly related to the ferroelectric transition. The maximum in *Q* ^−1^ occurs in a quite different temperature range and is not obviously related to the dielectric loss

The capacitance has a broad, frequency-dependent maximum centred on ~230 K at 100 Hz and ~235 at 1 MHz (Fig. 4e). There is then a steep increase at higher temperatures which also has a much stronger frequency dependence. This pattern is mirrored in the data for tan *δ* (Fig. 4f) which has a small peak just above 200 K, weakly dependent on frequency, and peaks at higher temperatures with much stronger dependence on measuring frequency. When plotted in an Arrhenius manner as ln*f* versus 1/*T*_m_ (Fig. 6), the capacitance data show some non-linearity, which has been fit using Eq. (1) to give Vogel–Fulcher parameters that are listed in Table 1. The weak frequency dependence of *T*_m_ and only very slight non-linearity suggest weak relaxor character. An Arrhenius treatment of the dielectric loss peak at higher temperatures gives an activation energy of ~0.7 eV and, following Raevski et al. [33], is attributed to conductivity. This is likely also to be the origin of anomalies in the other high-temperature data (Fig. 4g, h).

### PZTFT4

**Figure 7:**
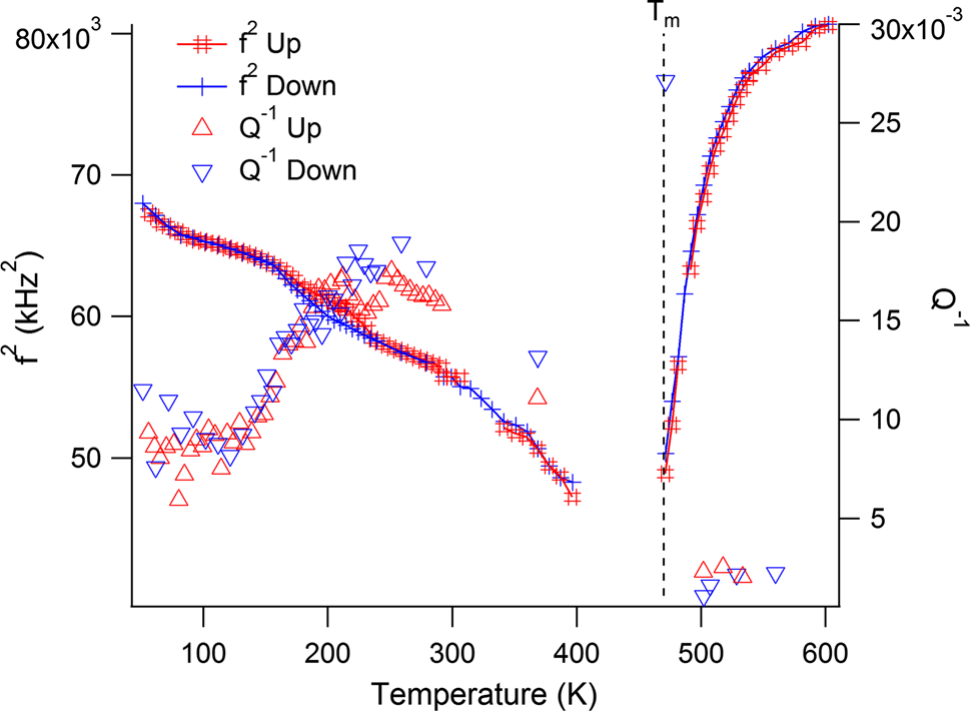
RUS data for PZTFT4 reproduced after Schiemer et al. [8]. Data are missing between ~400 and ~475 K because of strong attenuation in this temperature interval. It was possible to follow peak positions between 400 and 300 K but the peaks themselves were too weak to allow reliable determination of their widths for determination of *Q* ^−1^

RUS data for PZTFT4 are reproduced from Schiemer et al. [8] in Fig. 7 not only for comparative purposes but also for reinterpretation in the light of the results from PFT and PZT–PFN [16]. The onset of softening with falling temperature occurs somewhere above the highest temperature at which spectra were collected and the softening trend increases steeply below ~550 K. Resonance peaks disappear between ~475 and 400 K due to high attenuation. Recovery of the shear modulus (∝*f* ^2^) has a slightly irregular pattern with a clear hysteresis between cooling and heating occurring through the temperature interval 170–235 K. A relatively steep increase in *f* ^2^ occurs below ~50 K.

The sample used for RUS was the same as described by Sanchez et al. [12] for which dielectric and magnetic data have already been presented [8, 12, 61, 62]. There is a broad peak in the dielectric permittivity at ~470 K without any change in the temperature of the maximum as a function of measuring frequency. The capacitance and tan *δ* show a small frequency-independent peak at ~240 K in data collected during cooling [8], and the peak in capacitance also occurs at about the same temperature during heating.

The magnetic hysteresis loop measured at room temperature has a small opening, indicating the presence of some ferromagnetic component. FC and ZFC measurements of magnetic susceptibility diverge and show anomalies at ~50 and ~15 K [8].

### PZTFT3

RUS spectra collected from a fragment of [Pb(Zr_0.53_Ti_0.47_)O_3_]_0.7_[PFN]_0.3_ (PZTFT3), weighing 0.0019 g, showed a clear change in resonance frequencies between 213 and 218 K during cooling and between 232 and 237 K during heating, consistent with the hysteretic pattern seen in more or less the same temperature interval in PZTFT4. An intermediate temperature of 225 K is shown as marking the tetragonal–monoclinic transition in Fig. 2. The fragment was part of the original sample described by Sanchez et al. [12] which had a peak in dielectric permittivity at a frequency-independent temperature close to 530 K and weak ferromagnetism at room temperature. Sanchez et al. [12] also reported that it had a steep increase in magnetisation below 50 K. Due to the smallness of the sample, it was not possible to obtain further elasticity data in this temperature interval or at high temperatures.

## Discussion

### Binary phase relations

Although there are fewer data for lower temperatures, the binary Pb(Zr_0.53_Ti_0.47_)O_3_–PFT phase diagram is closely similar to that of Pb(Zr_0.53_Ti_0.47_)O_3_–PFN. Softening of the shear modulus starts ~150–200 K above the freezing interval of PFT and is presumed to occur at a similar temperature interval above the cubic–tetragonal ferroelectric transition. At intermediate compositions, the transition is accompanied by a broad maximum in the dielectric constant at a temperature which varies linearly with composition (within reasonable experimental uncertainty) and occurs ~30 K above the minimum in the shear modulus. The location of the weakly first-order tetragonal–monoclinic transition has not been independently determined but, by analogy with Pb(Zr_0.53_Ti_0.47_)O_3_–PFN, is taken as being marked by the hysteretic change in shear modulus between ~200 and ~250 K. No real evidence for a tilting transition has been found and there is no indication in the RUS data of magnetoelastic coupling. In contrast with the original interpretation of Schiemer et al. [8], the non-linear elastic stiffening at low temperatures is most likely due to anelastic changes associated with freezing of defects which have a spectrum of relaxation times. In PFN, at least, these appear to be related to the ferroelastic microstructure which had been reported to be tweed-like.

From the perspective of elasticity, the ferroelectric transition displays the classic influence of linear quadratic strain/order parameter coupling that defines improper ferroelastic behaviour. An applied stress induces a strain and this in turn induces a relaxation of the order parameter. In the idealised case of a second-order transition, this would induce a step-like softening by some constant amount. A more nearly tricritical evolution of the order parameter would lead to a non-linear recovery of the related elastic constants with falling temperature and the *f* ^2^ data for PZTFT4 are consistent with this (Fig. 7).

Comparison of the tetragonal strain values at room temperature for PZT–PFN with those of PZT–PFT in Fig. 3 shows the same reduction of shear strain associated with changing composition away from PZT but for PFT the effect is substantially greater. Even below 200 K in PFT, the values of *e*_t_ are less than the realistic limit of resolution from the primary diffraction data and the structure may be rhombohedral. This trend seems to correlate with the development of relaxor characteristics which also increase with increasing Fe/Ta disorder in PFT [34]. Causal relationships are not established but decreasing shear strain leads to lower strain contrast across twin walls or boundaries between PNR’s, and disordering generates strain heterogeneity at a unit cell scale. Both effects could favour short range order in place of long ranging correlations at a discrete cubic–tetragonal transition.

### Tetragonal–monoclinic transition

As in PZT–PFN, the tetragonal–monoclinic transition at intermediate compositions is taken as being marked by the hysteretic change in *f* ^2^. Although the transition is accompanied by the development of new shear strains, $e_{\rm{o}}^{'}$ and $e_{5}^{'}$, however, there is no additional softening of the form that would be expected from linear–quadratic strain/order parameter coupling and there is no sign of the increase in acoustic loss that would be expected to accompany the appearance of new ferroelastic twin walls. Rather, there is a small increase in elastic stiffness and a reduction of *Q* ^−1^ (Fig. 7). In other words, the normal relaxation which is evident at the cubic–tetragonal transition does not occur and there is a reduction in twin wall mobility under the stress and frequency conditions of RUS. This result is closely analogous to observations for (Ca, Sr)TiO_3_ perovskites in which the first tilting transition shows all the characteristics of improper ferroelastics, while these are suppressed at the appearance of the second tilt system [71, 72]. It is also analogous to the elastic stiffening which accompanies the ferroelastic phase transition in SrAl_2_O_4_ [73].

The common feature in these other cases is the operation of two discrete order parameter, and the result differs from a sequence of transition which involve only the reorientation of a single-order parameter. The latter is represented by the $Pm{\bar{3}}m$–*P*4*mm*–*Amm*2–*R*3*m* sequence of BaTiO_3_, in which each transition is marked by a minimum in the shear modulus [72]. If there is a general conclusion, it might be that coupling of strains between two separate order parameters results in some kind of jamming, both of the strain relaxation and the mobility of twin walls. This would imply that the monoclinic phase of PZT–PFN and PZT–PFT arises as a consequence of a second discrete instability rather than reorientation of the $\varGamma_{4}^{ - }$ order parameter alone.

### The influence of microstructure

The classical treatment of strain/order parameter coupling does not take account of the influence of microstructure, and improper ferroelastic transitions in perovskites appear to be invariably accompanied by additional softening effects associated with the development and evolution of ferroelastic microstructures (e.g. see review of literature data in [72]). Precursor softening is due to dynamical effects and, in the case of ferroelectric transitions, can be understood in terms of the development of polar nano-regions (PNR’s). The onset of softening in PbMg_2/3_Nb_1/3_O_3_ occurs at the Burns temperature, which itself marks the appearance of dynamical PNR’s (e.g. [74]). The onset of such softening for PFT and for other members of the PZT–PFT solid solution is likely to have the same origin (*T*_ps_ in Fig. 2).

Collective motions involving displacements of boundaries between PNR’s with local tetragonal symmetry or between the PNR’s and a cubic matrix would give rise to a central peak mode with relaxation rather than phonon characteristics. If the relaxation time for the collective motion of local domains increases as the ferroelectric transition is approached from above, it is inevitable that the acoustic modes which couple to it will also soften. In the case of PFN, evidence that some part of the dynamical microstructure becomes quasi-static at a temperature above the transition point was provided by a small increase in acoustic loss [20], and a similar feature perhaps exists at ~275 K in the data for PFT (Fig. 4a).

The difference between *T*_m_ and the temperature at which the minimum in shear modulus is reached presumably also arises as a consequence of the development of microstructure. The dielectric maximum relates to the development of longer ranging correlations of ferroelectric dipoles, but the development of ferroelastic twins is not necessarily the same because of a difference in length scale for strain fields and polar interactions. The elastic constants measured in an RUS experiments are those of the whole sample and are therefore some average over all the domains and twin walls present. However, the thickness, *w*, and number density, *N*, of ferroelastic twins are expected to diverge at the transition point as *w* ∝ *N* ∝ (*T*_c_−*T*)^−1^ [75–78] and the measured elastic properties will only become those of the ordered domains at some temperature below *T*_c_ when the twin walls reduce in volume to only a small fraction of the total volume of the crystal. It appears that *T*_m_ is just above the transition temperature, *T*_c_, and the rounded minimum in elastic constants would occur just below this.

In a classic improper ferroelastic perovskite, such as LaAlO_3_, anelastic losses are due primarily to the mobility under stress of the ferroelastic twin walls [79–81]. The expected pattern is a steep increase at *T*_c_ when thick, highly mobile twin walls first appear, and a broad maximum immediately below *T*_c_ depending on the changing number density and increasing interaction of thin walls with defects. A plateau of relatively high loss then defines a temperature interval in which thin twin walls move in an effectively viscous medium. This ends with a Debye loss peak when the walls become immobile due to pinning by defects such as oxygen vacancies. The pattern of high loss immediately below the ferroelectric transition and the plateau of high loss are seen in PZTFN4 (Fig. 6) but, just as in PZT–PFN, *Q* ^−1^ remains high down almost to the lowest temperatures. In place of the discrete freezing interval for twin wall motion, there appears to be freezing of defects with a spectrum of relaxation times.

### PFT

Although superficially similar, the transformation behaviour of PFT is different in detail from that of both PFN and intermediate members of the PZT–PFN/PFT solid solutions. Firstly, in place of a discrete cubic–tetragonal transition, frequency-dependent peaks in the capacitance at ~220–235 K are indicative of relaxor character and there is no evidence for distortion from cubic lattice geometry other than very weak and continuous changes in birefringence. Secondly, measurable spontaneous strains appear only below the weakly first-order transition to a monoclinic or rhombohedral structure at ~205–210 K (Fig. 1; [24]). Steep softening of the shear modulus is closely similar to what is seen at other compositions and is typical of the influence of linear/quadratic strain/order parameter coupling and PFT also shows a narrow, discrete peak in *Q* ^−1^ at 210 K. Variations in the heat capacity also show two quite different effects [44, 45], the first being a broad excess in the interval ~230–325 K, which must be due to the relaxor freezing, and the second being a sharp peak at 205 K, which coincides with the first-order transition. Thirdly, the broad peak at ~150 K is typical of the acoustic loss associated with freezing of ferroelastic twin wall motion and occurs in place of the plateau in loss seen for PFN and intermediate members of the solid solutions.

From the perspectives of strain and elasticity, the only transition occurs at ~210 K and seems to have all the attributes of being improper ferroelastic, with twin wall mobility continuing down to ~150 K. There is a hysteresis in *f* ^2^ between heating and cooling (Fig. 4a), with an upper temperature limit of 210 K at 160 kHz and 240 K at 400 kHz, as would be expected if there is an irreversible coarsening of ferroelastic twins during cooling. The origin of a small jump at 210 K (160 kHz) or 240 K (400 kHz) is not so clear but presumably relates to the known first-order character of the transition. The Arrhenius plot (Fig. 6) seems to confirm that there are few quantitative correlations between the dielectric (relaxor) and elasticity (improper ferroelastic) data.

The minimum in shear modulus is rounded and occurs at ~180 K rather than at the transition temperature, ~210 K. A rounded minimum had been seen previously in ceramic samples by Blazhevich et al. [41], Smirnova et al. [55] and Smirnova et al. [56], and can be attributed to the influence of ferroelastic microstructure. The same overall pattern as reported here for precursor softening, softening through the transition, and the Debye-like peak in acoustic loss centred on ~140 K were also seen in the velocity of longitudinal waves at 10 MHz, which depends on both the shear and bulk moduli [55, 56]. The ultrasonic data show additional subtle variations in attenuation at ~180 and ~35 K, which perhaps reflect contributions of magnetic ordering to anelastic changes in the bulk modulus. Fitting of the broad peak in *Q* ^−1^ as a single Debye loss peak, following the procedure used for similar peaks in RUS data from other perovskites [72], gives a low activation energy (~0.035 eV) but there are multiple overlapping peaks and, therefore, more than one strain relaxation involved.

The view of PFT as having two separate structural responses to changing temperature is consistent with the suggestion also for PFN [20] and PZT–PFN [16] that the monoclinic structure occurs as a result of a second discrete instability, rather than simply the reorientation of the ferroelectric dipole that occurs in BaTiO_3_. The first instability is predominantly ferroelectric (relaxor in PFT) and the second is predominantly ferroelastic. In BaTiO_3_, each of the transitions derived from changing orientation of a single order parameter is marked by a peak in the dielectric constant [82], while there is no evidence that the second instability in PFT has any influence on the dielectric properties (Fig. 4e; Refs. [19, 28, 32, 37, 38, 43]).

If the precursor softening is due to a dynamic microstructure which slows down as the freezing point is approached, it might conform to a description as Vogel–Fulcher freezing according to

2 $$ \Delta C\; \propto \;\exp \left( {\frac{{ - E_{\text{a}} }}{{k_{\text{B}} (T_{\text{m}} - T_{\text{VF}} }}} \right) $$

**Figure 8:**
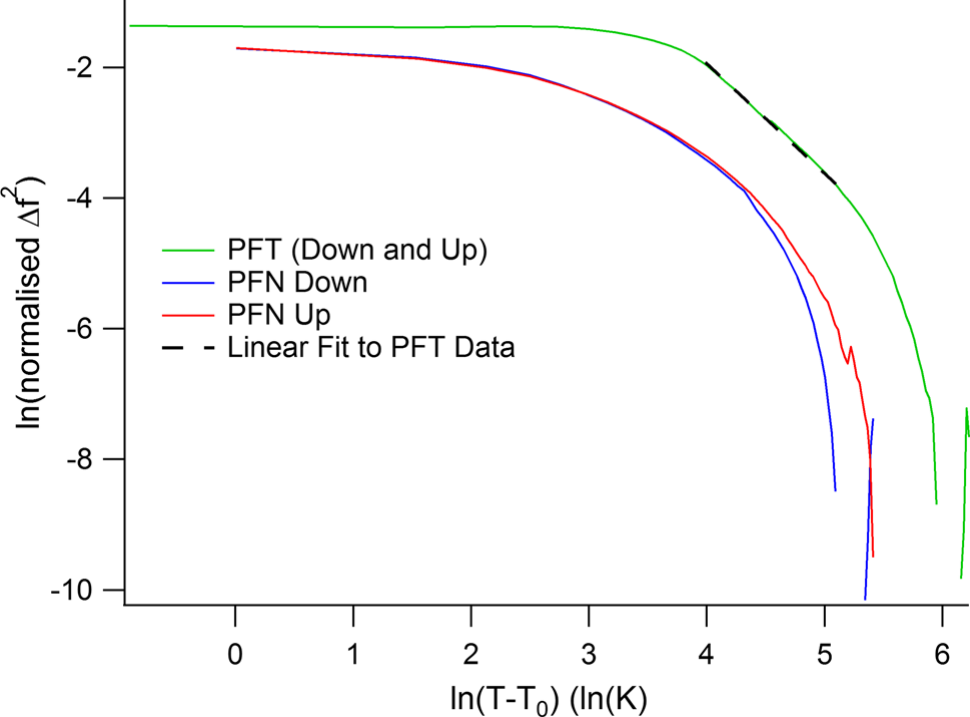
Variations with temperature of precursor softening in PFT and PFN. Δ*f* ^2^ is the difference between observed values of the square of the resonance frequency for individual RUS peaks and a linear baseline fit to the highest temperature values in the stability field of the cubic phase. These have been normalised to 1 at 605 K for PFT and 610 K for PFN. The *dashed line* is a fit of Eq. (3) to data for PFT between ~250 and ~400 K with *T*_c_ set at 200 K. *T*_c_ was set at 380 K for PFN

The softening described in this way for PbMg_1/3_Nb_2/3_O_3_ has an effective activation energy of ~0.01 eV [74]. If the softening is due to elastic fluctuations related to a soft optic mode, it would be expected to follow a power law of the form:

3 $$ \Delta C\; \propto \;(T - T_{\text{c}} )^{ - \kappa } , $$

as shown for example in the case of LaAlO_3_ [83]. The exponent, *κ*, is expected to have values between 1/2 and 2, depending on the dispersion of the soft mode in three dimensions (e.g. [84]). In order to test these alternative descriptions, a linear baseline was fit to the highest temperature data for *f* ^2^ of PFT and the softening was taken to be the difference, ∆*f* ^2^, between observed values and the baseline extrapolated to lower temperatures. There appears to be no combination of parameters which allows Eq. (2) to represent the temperature dependence of ∆*f* ^2^ but, as shown in Fig. 8, there is a limited temperature interval between ~250 and 400 K over which the power law (Eq. 3) provides a fit, with *κ* ≈ 1.7 for *T*_c_ = 200 K. The change in trend below ~250 K is understandable because it coincides with a steep increase in acoustic loss while data at the highest temperatures are for small values of ∆*f* ^2^ that are most sensitive to the choice of baseline. A value of *κ* = 3/2 would imply softening along one branch of the soft mode, as discussed at greater length for LaAlO_3_ [81].

Precursor softening in PFN does not fit with either the Vogel–Fulcher description or a power law (Fig. 8). This aspect of the precursor dynamics is not resolved here but perhaps the observed softening is due to a combination of relaxational contributions and fluctuations of the type implied by Eq. (3). The relative contributions then differ for PFN which has a discrete ferroelectric transition with measurable tetragonal shear strain and, most likely, a precursor tweed microstructure, in comparison with PFT which is relaxor without measurable shear strain.

Electron paramagnetic spectra suggest that there is an increase in short range magnetic correlations below ~350 K in PFT, which is in the same range as the precursor elastic softening [85]. No evidence has been found for a discrete ferromagnetic transition, however, and ferromagnetism at room temperature in the sample described here could be due in part, at least, to defects or the presence of an impurity phase. The magnetic data also do not provide conventional evidence for antiferromagnetic ordering at low temperatures, but the inflection in magnetic moment under field-cooled conditions at ~170 K (Fig. 4b) occurs within the expected temperature range. Such a high value of *T*_N_ would be consistent with a relatively high degree of B-site disorder according to the dependence found by Kubrin et al. [34]. There is no evidence in the elasticity data for any associated anomaly in the elastic properties, but if the strain accompanying the magnetic ordering is only due to a change in volume, this would be expected to be seen in data for the bulk modulus. RUS data are representative, predominantly, of the shear modulus. Similarly, there is no evidence of magnetoelastic coupling in the RUS data near 50 K where the magnetisation measured under field-cooled conditions increases (Fig. 4c; [40]) other than, perhaps, a small break in slope of *Q* ^−1^. This change in magnetic order, as well as the possible development of spin glass near 10 K, occurs within a temperature interval where the shear modulus is increasing steeply with falling temperature, however, and, as with the high-temperature short range ordering, such changes in magnetic properties may be a reflection of final adjustments or freezing of the ferroelastic microstructure.

### Strain heterogeneity

The microstructure of PZTFT4 at room temperature is known from characterisation by transmission electron microscopy [86]. Its most distinctive features are nested domains with curved boundaries on length scales of ~10–100 nm, together with strong contrast which implies that there is significant local strain across them. 180° walls are ferroelectric and would not be expected to show strain contrast, while 90° walls are both ferroelectric and ferroelastic—and it is presumably these that are seen in the electron micrographs. This microstructure is quite different from what would be expected in a ferroelastic material with large symmetry breaking shear strain, i.e. well-defined twin domains with planar interfaces. Evans et al. [1] also showed that the nanoscale microstructure can self-organise into other configurations under the influence of an externally applied magnetic field. The same must apply in response to an applied stress field and the mechanism for acoustic loss will involve motion of the curved boundaries. With such local heterogeneity, it is likely that different parts of the twin walls will relax with a spectrum of relaxation times, rather than the single relaxation time associated with a single mechanism such as motion of ledges along discrete, thin, more nearly planar walls. As discussed also for PFN [20], this is consistent with the wide temperature interval of high loss down to low temperatures and the non-linear anelastic stiffening represented by the upward curvature of *f* ^2^ with falling temperature. The effect of the transition to a monoclinic structure on the nested domain microstructure is not known but appears to result in a drop in the twin wall mobility.

In addition to the mesoscopic length scale of strain heterogeneity indicated by the ferroelastic microstructures, diffuseness of the ferroelectric transition at intermediate compositions in the solid solution points to heterogeneities at a unit cell scale. The diffuseness increases with increasing size disparity of substituting cations substituted in PFN [86] and must originate, at least in part, from local strain fields generated by even small amounts of the substitution of Fe^3+^+Ta^5+^ for Ti^4+^+Zr^4+^. The ionic radius of Fe^3+^ ($0.55 \,\AA$) is more than 20 % smaller than that of Zr^4+^ ($0.72 \,\AA$). Based on results from the (La,Pr)AlO_3_ solid solution [87], the length scale of strain fields around Fe^3+^ as an impurity in PZT is likely to be ~$15{-}20 \,\AA$. In the case of an octahedral tilting transition in La_0.6_Sr_0.1_TiO_3_, local strain heterogeneity accompanying disordering of Sr and vacancies on the crystallographic B-site causes the macroscopic spontaneous strain to almost disappear without suppressing the phase transition itself [88]. The same local heterogeneity would contribute to the drop in macroscopic shear strain coupled to the cubic–tetragonal transition away from the PZT end member of PZT–PFT (Fig. 3). In other words, local strains impede the development of long ranging strain coupling with the ferroelectric order parameter.

The nature of changes in magnetic properties near 50 K and below ~15 K described by Sanchez et al. [12] and Schiemer et al. [8] is not fully understood, but they clearly occur in a material with substantial local strain heterogeneity and complex ferroelastic microstructures. If there is any coupling between magnetisation and strain, it is inevitable that this will influence the manner in which magnetic moments become aligned on the same length scales.

## Conclusions

The location of the ternary MPB in the system PZ–PT–PFT is not known but the binary phase diagram for PZT–PFT is closely similar in form to that for PZT–PFN, and the pattern of elastic and anelastic anomalies for PZTFT4 is also similar to that seen for PZTFN4. There appear to be two discrete instabilities which couple with strain, but no direct evidence for any coupling of magnetic ordering with shear strain. In contrast, the behaviour of PFT has significant differences from that of PFN. Much weaker coupling of the tetragonal shear strain is associated with the change to relaxor character for the ferroelectric properties, but the second transition, either to a monoclinic or rhombohedral structure, has the attributes of being classical improper ferroelastic. As with PZT–PFN, the form of stiffening and acoustic loss at low temperatures is consistent with the existence of heterogeneous strain at both mesoscopic and microscopic length scales. These are likely to be pervasive, more generally, in multicomponent multiferroic perovskite solid solutions.
